# Factors contributing to nass consumption among Iranian Turkmen: A qualitative study

**DOI:** 10.18332/tid/93697

**Published:** 2018-08-27

**Authors:** Shirin Shahbazi Sighaldeh, Abdurrahman Charkazi

**Affiliations:** 1 Reproductive Health Department, School of Nursing and Midwifery, Tehran University of Medical Sciences, Tehran, Iran; 2 Environmental Health Research Center, School of Health, Golestan University of Medical Sciences, Gorgan, Iran

**Keywords:** smokeless tobacco, nass, contributing factors, Turkmen, qualitative study

## Abstract

**INTRODUCTION:**

Nass is a smokeless tobacco product. Iranian Turkmen have a long history of nass consumption. However, the factors contributing to nass consumption among Iranian Turkmen are not known. The purpose of the present study is to examine the factors contributing to nass consumption among Iranian Turkmen.

**METHODS:**

This qualitative study was conducted between January and March 2016 in four Turkmen cities of Golestan province in Iran. Participants included 34 male Turkmen nass consumers. Data were collected through individual and group interviews and were analyzed by content analysis. Data management was done by qualitative data analysis software MAXQDA, Version 10.

**RESULTS:**

The results of data analysis revealed the following as the main reasons for nass consumption by the study population: 1) cultural, social, and environmental facilitators, 2) nass was considered as an alternative to cigarette smoking, 3) nass was believed to intensify the effects of opium and other drugs, 4) specific occupations and circumstances, and 5) beliefs related to nass.

**CONCLUSIONS:**

Cultural and historical backgrounds, convenient access to nass at a very low price, curiosity, emulation, and peer pressure were the main factors driving nass consumption among Iranian Turkmen. Various beliefs, such as the idea that nass intensifies the effects of opium and alcohol, calms the nerves, and helps individuals quit smoking were also found to contribute to this phenomenon. Finally, individuals in certain lines of work, such as fishing, driving combines, and military service, were more likely to consume nass.

## INTRODUCTION

Many forms of smokeless tobacco are used around the world. For example, chewing tobacco and moist snuff are used in United States, and Snus is consumed in Sweden^1^. Like other forms of tobacco, smokeless tobacco contains nicotine, which is an addictive substance^1^. With policies set to promote clean air in public places and to reduce the effect of tobacco smoke, the use of smokeless tobacco in the USA and Scandinavian countries has gained popularity in recent years^2^. On the other hand, smokeless tobacco products are widely used by people who need help to quit smoking^3^. However, this product has its own detrimental effects on an individual’s health, and has caused serious debates among medical and health professionals^4^.

**Figure 1:**
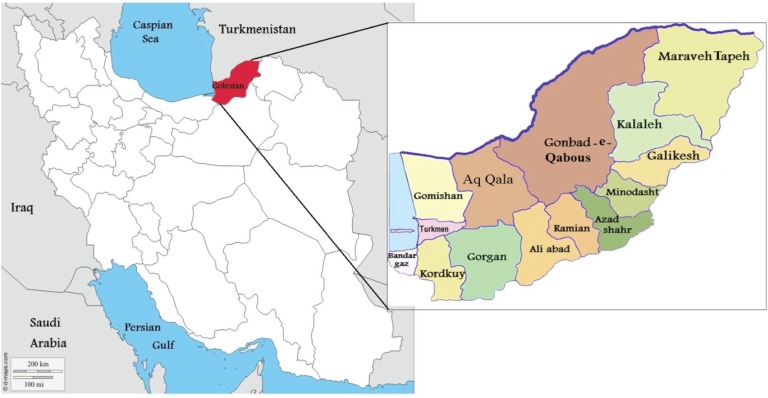
Geographic map of the study area

**Figure 2:**
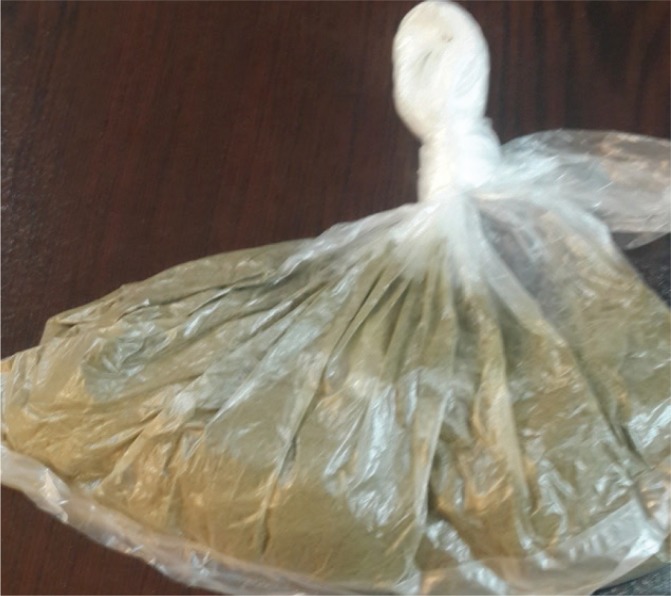
Bulk form of Turkmen nass. Turkmen nass includes tobacco mixed with ash, salt and water. It is usually consumed by the elderly. Turkmen nass is less expensive and has a lighter color than Afghan nass.

**Figure 3:**
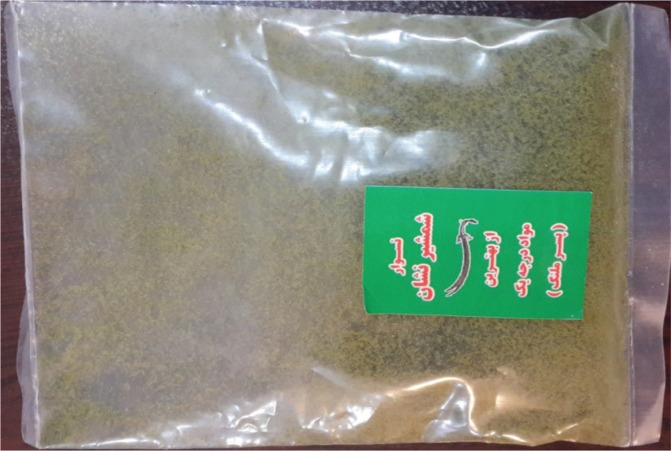
Pesare Malang nass in its original packaging. Pesare Malang is one of the most famous types of Afghan nass and comes in 50 g plastic packages. This brand is more popular among young nass consumers. It is made by mixing ground tobacco with lime, salt, water, and pepper

**Figure 4:**
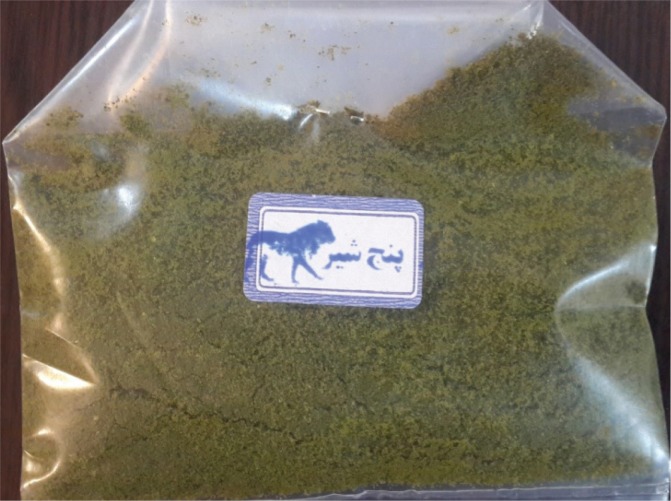
Panjshir nass in its original packaging. The word Panjshir means ‘five lions’. Panjshir is a bulk form of Afghan nass that is used in some regions of Golestan province, specifically in Gonbad-e-Qabous. It is composed of ground tobacco, lime, salt, and water. It comes in plastic packages of approximately 50 g. It is more commonly used by younger adults.

In Central Asian countries, such as Iran, Afghanistan and Pakistan, smokeless tobacco is known as nass and contains a mixture of tobacco leaves and substances such as lime and ash^5^. Iranian Turkmen live in the two northern and north-eastern provinces of Golestan and North Khorasan of Iran. The Golestan Province in situated in the north of Iran and south-east of the Caspian Sea. According to the 2016 census, the population of the Golestan province was about 1.7 million, of which about 40% were Turkmen^6^. The province of Golestan includes 14 cities (Figure 1). The Turkmen ethnic group in the Golestan province lives in its central and northern areas, known as Turkmen-Sahra (Turkmen steppes). Turkmen have a long history of nass consumption, and sublingual use of nass is common among them. Turkmen keep nass between their gums and lower lip for 2 to 10 minutes before discarding it. In an effort to make it more hygienic, some people wrap the nass in a paper tissue and place it under their tongue; in this case it takes longer for nass to have an effect. The nass used by Turkmen comes in 50 g plastic packages. There are two types of nass: Turkmen nass and Afghan nass. Elderly men are the main consumers of Turkmen nass, made by combining ground tobacco with ash, salt, and water. They have learned how to make nass from their ancestors and they make nass in their own small workshops at home. Turkmen nass (Figure 2) has a lighter color than Afghan nass. Afghan nass is more popular among young consumers, and it is also produced in the province. Afghan nass is made by mixing ground tobacco with lime, salt, water, pepper, and other synthetic materials. Afghan nass comes in plastic packages under brand names such as Pesare Malang and Panjshir (Figures 3 and 4). The production of nass (both Afghan and Turkmen) is illegal. Individuals who make nass do not have a production license and such activity is not supervised by officials. Therefore, the amount and type of materials used in making nass is unclear. Also, unlike different types of smokeless tobacco used in Western countries, nass is not pasteurized and its nicotine concentration is uncertain. While in the past consumption of nass was more common among older people, these days it is widely used by young adults too. Examining the results of a cohort study on 50045 people aged 40–75 years, Islami et al.^7^ found that 42.4% of the participants were male, 1.1% smoked hookah and 7.5% consumed nass.

Qualitative research provides researchers with valuable and critical information of participants and their thoughts and feelings. Qualitative studies examining the factors contributing to nass consumption allow direct interaction with real consumers and their opinions and experiences. The data collected in such studies reflect the reasons for nass consumption more accurately^8^. Based on our literature review, the prevalence of nass and the factors contributing to nass consumption among Iranian Turkmen have not been examined before. Therefore, the purpose of the present study was to provide a thorough understanding of nass consumption and its contributing factors in these people.

## METHODS

This qualitative descriptive study was conducted during Winter 2016 in four major Turkmen cities of Golestan province: Gonbad-e-Qabous, Aq Qala, Ghomishan, and Bandar-e-Turkmen. Thirty-four nass consumers of Turkmen ethnicity participated in this study. Participants were 17–70 years old and were selected from different areas of Turkmen-Sahra. Purposive sampling was used to allow maximum variation in terms of age, location, occupation and marital status (Table 1). Every participant was interviewed separately. Furthermore, three focus group discussions were carried out, with 4 or 5 participants in each group. The discussions were carried out unplanned and in natural environments (Table 2).

**Table 1:** Participants characteristics

*Participant’s ID Number*	*Age*	*Occupation*	*Marital status*	*Education*	*Age (years) when nass was first used*	*Place of residence*	*Smoking status*
1	31	Plumber	Married	High school dropout	20	Aq Qala	Ex-smoker
2	32	Laborer	Married	Secondary school	19	Aq Qala	Ex-smoker
3	32	Unemployed	Married	Secondary school	19	Aq Qala	Ex-smoker
4	28	Laborer	Single	Primary school	22	Aq Qala	smoker
5	37	Unemployed	Married	Secondary school	16	Ghomishan	Ex-smoker
6	50	Fisherman	Married	Secondary school	35	Ghomishan	Smoker
7	55	Fisherman	Married	Primary school	45	Ghomishan	Ex-smoker
8	35	Fisherman	Married	Secondary school	14	Ghomishan	Smoker
9	18	Shepherd	Single	Secondary school	12	Ghomishan	Non-smoker
10	60	Unemployed	Married	Illiterate	15	Ghomishan	Smoker
11	60	Unemployed	Married	Illiterate	48	Ghomishan	Non-smoker
12	23	Laborer	Single	Secondary school	18	Gonbad-e-Qabous	Ex-smoker
13	27	Laborer	Married	High school diploma	14	Ghomishan	Non-smoker
14	22	Laborer	Single	Primary school	17	Aq Qala	Non-smoker
15	57	Farmer	Married	Primary school	35	Gonbad-e-Qabous	Ex-smoker
16	50	Driver	Married	Associate degree	45	Ghomishan	Ex-smoker
17	50	Farmer	Married	University degree	16	Ghomishan	Non-smoker
18	18	Unemployed	Single	Secondary school	12	Turkmen	Non-smoker
19	32	Skilled worker	Married	High school diploma	14	Turkmen	Ex-smoker
20	30	Skilled worker	Married	High school dropout	14	Turkmen	Ex-smoker
21	34	Farmer	Married	High school dropout	15	Turkmen	Non-smoker
22	28	Unemployed	Married	Primary school	14	Turkmen	Non-smoker
23	52	Shopkeeper	Married	Secondary school	39	Turkmen	Ex-smoker
24	36	Farmer	Married	Primary school	22	Gonbad-e-Qabous	Non-smoker
25	21	Unemployed	Single	Primary school	17	Aq Qala	Non-smoker
26	34	Driver	Married	Secondary school	19	Gonbad-e-Qabous	Non-smoker
27	28	Driver	Married	High school dropout	19	Gonbad-e-Qabous	Non-smoker
28	21	Skilled worker	Single	Primary school	18	Aq Qala	Non-smoker
29	27	Skilled worker	Single	High school diploma	24	Aq Qala	Smoker
30	22	Skilled worker	Single	High school dropout	17	Aq Qala	Non-smoker
31	22	Skilled worker	Married	Secondary school	18	Aq Qala	Non-smoker
32	60	Retailer	Married	Unemployed	53	Aq Qala	Ex-smoker
33	70	Farmer	Married	Primary school	12	Aq Qala	Ex-smoker
34	70	Farmer	Married	Illiterate	40	Aq Qala	Ex-smoker

**Table 2:** Participants in the focus group discussions

*Number of participants*	*Place of interview*	*Town*
4	Aluminum workshop	Ghomishan
5	Inside a shop	Bandar-e-Turkmen
4	Home	Aq Qala

### Data collection

Data were collected through individual semi-structured interviews and focus group discussions. The main question was: ‘what triggers the consumption of nass?’. Three focus group interviews were also carried out to examine group, social and cultural dynamics of participants in their natural environments^9^. Interviews were done in public places, such as in front of shops. Interviews were continued until data saturation was reached (no new information was obtained). All interviews were carried out by the first author who is a Turkmen. Interviews were done in the Turkmen language. On average, each interview took half an hour; and each focus group discussion lasted an hour. Before each interview, the purpose of the interview was explained to the participants and they were assured that the interviews will not have any negative effect on them. The participants were also told that they did not need to disclose their names and could terminate the interview and withdraw from the study at any time without being asked for a reason. Participants were assured that the collected information would be kept confidential. All interviews were audio recorded and transcribed to text in the Turkmen language, as soon as possible. Upon completion of the interview, participants were given a mobile phone charge worth 50 thousand Rial (about US$0.5) as a token of appreciation for their participation in the study.

This study, with the project number: 9504010770 and code of ethics IR.GOUMS.REC.1395.86, was approved by the Ethics Committee of the Research and Technology Department of the Golestan University of Medical Sciences.

### Data analysis

Themes contributing to nass consumption were extracted through conventional content analysis. Statistical software MAXQDA, Version 10, was used to manage the codes. To avoid loss of meaning through translation, analyses were done in the Turkmen language.

## RESULTS

The results showed that the following were the main reasons for nass consumption: 1) cultural, social, and environmental facilitators, 2) nass was considered as an alternative to cigarette smoking, 3) nass was believed to intensify the effects of opium and other drugs, 4) specific occupations and circumstances, and 5) beliefs related to nass.

### Cultural, social and environmental facilitators

Cultural background was described as one of the factors contributing to nass consumption. People become familiar with nass as they watch their elders use it. One of the participants stated: *“nass has traditionally been here and people have been using it. We see our elders use it and we learn from them. Youngsters see it in their elders’ hands and they learn.”* (Participant No.12 or P.12).

Easy and convenient access to nass was another contributing factor to its consumption. In those areas, one could easily find nass in any store. Another participant said: *“You know, it is very accessible. Wherever you go you can see it in shops [and you can buy it] without any restrictions. It is easy to obtain. For example, you do not need to go to a dealer’s house or see anyone else. It is easy to get. A Japanese proverb says ‘for a Samurai, everywhere is Japan’, we say ‘for a nass user, nass is everywhere’.”* (P.22).

When non-users of nass frequently interact with nass consumers, they become curious. Easy access to nass and the sense of curiosity due to frequent observation of its use by others could contribute to initiating nass consumption. *“This is Turkmen-Sahra and all Turkmen use nass. You see it in the hands of others and you become curious. So you say to yourself, let’s try it to see what it is. That’s how I started using it.” (P.24)*.

The lower price of nass compared to cigarettes also boosts people’s tendency to start using it. On the other hand, a pack of nass lasts much longer than a pack of cigarettes; therefore, it is more economical to use nass. *“A pack of nass is 500 Toomans, which is more economical than cigarettes, right? You buy a pack of cigarettes for two thousand Toomans and that may last for a day. But a package of nass that you buy for 500 Toomans may last you for three to four days.”* (P.3).

Offering nass to friends is a common practice among Turkmen. Some participants claimed that they started using nass since it was offered to them by their friends: *“Suppose someone is using it now and tells you ‘come on do you want to use some? Take some’. This is a common practice among Turkmen, and is like a tradition. Even if you have used some a few minutes ago, they say ‘come on, let’s use some together’. Nass is not something strange among Turkmen and they recommend it to each other.”* (P.32).

Also, when one starts using nass he may initially suffer from gastrointestinal problems, such as nausea and vomiting. Other common side effects are confusion and severe dizziness. Therefore, some experienced nass consumers tell adolescents and young people, who have not used nass before, that consumption of nass is not for everyone, and if they use it they won’t be able to tolerate the side effects. Sometimes, instead of discouraging, such comments challenge the young people to prove their strength and endurance, hence they start using nass to show that they are capable to do so. *“In our village there is a shop which is known as a distribution center for nass. When you use nass for the first time, it makes you dizzy. Kids gather in front of the shop and bet on who can keep Afghan nass under his tongue for a longer time. So whoever keeps it in his mouth longer is the winner and the looser must fill the belly of the winner. It means that, the winner eats whatever he wants from the shop and the looser must pay for it.”* (P.1).

Social factors, such as high unemployment rate and lack of proper recreation facilities in the area, were among other causes of nass consumption. “I say that, unemployment and lack of healthy recreation centers are the reasons for nass consumption. I think it is unemployment, because they are all unemployed and hang around here and there. Especially in villages like our village people are more likely to use it. You know, in big cities, you are busy with this and that. There may be nass in cities too, but it is less than here. Here, its percentage is too high.” (P.10).

Lack of crackdown and restriction by the government, on production, supply and use of nass, was considered another factor facilitating nass consumption. *“We do not have a proper government. If it was a decent government, it would have arrested all these opium dealers and put them in jail. It would have stopped all these new drugs and crimes. This is not the country of Turkmenistan; over there they quickly arrest the dealers and put them in jail. In Turkmenistan who can show nass to a government official?! They punish you. The use of nass and cigarette is forbidden, never mind opium and spirit; if they catch you with them, you will be punished. They do not allow you to use nass and cigarettes.”* (P.26).

### Alternative to smoking

Many participants said they use nass to help them quit smoking cigarettes. They mentioned the health of their families and friends as their reason for using nass instead of cigarettes. *“I used to smoke cigarettes but I can’t smoke anymore because of my wife and children. You are the one who smokes, but it harms the people around you. If you draw on a cigarette and send half of its smoke into your lungs and exhale the other half, that is something. But if with every draw on your cigarette, you exhale all the smoke out, that is something else; that will harm your wife and children significantly.”* (P.7).

Lack of odor was another reason for consumption of nass. The participants considered the smell of cigarette smoking very annoying. Nass, however, does not have any odor or its smell is negligible. *“I use it because it does not smell like a cigarette; that smell that stays with you anywhere you go.”* (P.19).

According to the participants, unlike cigarette, nass consumption does not require special preparation. Also, it can be used indoors. Nass can be consumed very quickly, in a matter of a few seconds, and it does not attract others’ attention like cigarettes do. One participant stated: “Using nass is much easier than using cigarettes because when you smoke a cigarette everybody can see you. For people like me, who do not smoke in front of their fathers and uncles out of respect for them, nass is a very good alternative. I personally know some people in the village who use nass and almost no one is aware of that.” (P.3).

### Intensifying the effect of opium and other drugs

Simultaneous addiction encourages nass consumption. Participants believed that consumption of nass after taking opium, alcohol, or Tramadol, intensifies the effect of these substances, sometimes doubling or tripling their effect and the accompanying pleasure. *“Because when you take drugs your mouth dries up and nass remains in your mouth longer and does not melt. Yeah, when you use nass after drug, it gives you a certain feeling; it’s like you are floating. In other words it doubles the euphoria.”* (P.10).

Believing that nass prolongs the effect of opium and causes the euphoria to last longer was another contributing factor mentioned by the participants: *“You know, the use of nass doubles the effect of opium. Usually one or two hours after the use of opium its effect starts to wear off. To overcome this, people use nass. They do this twice or three times until opium completely wears off and they have to use it again.”* (P.3).

### Special occupations and job environments

Participants claimed that certain occupations and job environments, including fishing, truck drivers and military service, encourage nass use. Illegal and unlicensed fishing from the Caspian Sea is common in those areas. *“The job of most people here is fishing, [where] they work in the sea. You cannot smoke cigarette when you have gone fishing illegally, you will get spotted and caught because of the cigarette light. Instead, they use nass which is smokeless and reduces the chance of being caught. For example, you are in a boat waiting for fish to catch, if you light a cigarette, you will be spotted. So you tell yourself ‘I cannot smoke cigarette till morning, so let’s use some nass instead.’ If you light your cigarette in the night, you will be captured by the guards.”* (P.8). Many people had started consuming nass while on military service. They believed that nass keeps you warm and helps you stay awake at night. *“When it was my turn to guard at night time, I put some nass in my pocket before heading to my post. When you use nass, particularly in cold weather, you feel stronger against cold. It also keeps you awake and more alert.”* (P.27).

Nass consumption was very common among truck drivers and their helpers. In those areas, the weather is warm during the harvest season of June and July. Therefore, truck drivers and their assistants prefer to use nass instead of cigarettes to prevent the potential danger of fire caused by careless use of cigarettes or improperly discarded cigarette butts. *“I started using nass when I became a truck driver and during the first harvest season. Well, I was smoking prior to that time and people advised me not to smoke while driving a combine because it may cause fire and burn the products. So, my colleagues suggested that I use nass instead and that is how I started using nass.”* (P.16).

### Beliefs related to nass

Some people had strong views and beliefs about nass, including its remedial effects; and such beliefs had motivated them to start using nass. For instance, nass is recommended by some older people as a remedy for toothache. *“I was a teenager, about 12 or 13 years old, and one night I had a severe toothache and my grandfather, who was a nass user, gave me nass to stop my toothache.”* (P.12).

Some participants believed that nass gives them the feeling of mental relaxation and comfort, and considered that to be the reason for starting nass consumption. *“I learned this while serving in the military. You are constantly nervous and stressed when serving in military. Whenever I used nass, I felt calm and my nervousness and agitation disappeared. Those were tough times and I was constantly nervous. When you take nass, you feel free and your pain disappears. So I started using nass to keep myself calm and relax.”* (P.12).

Some young people believed that using nass increased their physical strength, and that encouraged them to use nass. “I was working on a construction site with my friends. One of my friends suggested we use nass to boost our physical strength. That’s how I started using it.” (P.14).

## DISCUSSION

This qualitative research examined the factors contributing to nass consumption among the Turkmen ethnic group residing in Golestan province. The results showed that ‘cultural, social and environmental facilitators’ were one of the main factors contributing to nass consumption. Turkmen have a long history of using nass to the extent that Magtymguly Pyragy, a famous Turkmen poet and mystic (1724–1807), in one of his poems denounced and condemned consuming nass. Such evidence further confirms that using nass has a historical and cultural root among the Turkmen. In their study on the prevalence of smokeless tobacco in Myanmar, Sein et al.^10^ noted the historical and cultural background of chewing Kun-Yar. They also reported that the culture of chewing Kun-Yar had been mixed with people’s social customs and religious rituals and its related vocabulary had been included in primary and secondary school textbooks^10^.

Mass supply and easy access to nass at a very low price were among important environmental factors facilitating the use of nass, as mentioned by all participants. Unlike other drugs, nass can be bought easily and at a low price from almost every grocery store in Turkmen-Sahra. One does need to know a dealer. A 50 g pack of Afghan nass cost 500 Toomans, equal to 0.13 USD. Turkmen nass is even cheaper, it costs half as much as Afghan nass. In Myanmar, betel is offered in all public places for a very low price and there are no policies in place to prevent its sale to children under 18 years of age^10^.

The younger participants reported peer pressure as another factor contributing to nass consumption. Moreover, the availability of nass in the community created a sense of curiosity among adolescents and young people. They became curious about what nass offered that everyone was using it, and they decided to find out the answer through experience. This finding is in line with reports by numerous previous studies that have found direct and indirect peer pressure as one of the main contributing factors to smoking cigarettes and hookah^11-13^.

High unemployment rates among young people and lack of healthy recreational activities were among other factors contributing to nass consumption, as mentioned by a number of participants. Unemployed youth are easily attracted to non-healthy recreational activities such as smoking and using nass and other drugs.

Nass was considered as an ‘alternative to cigarettes’ by many participants. Participants claimed that they had decided to quit smoking due to health-related issues or complains from family members. Since there are no smoking cessation or counseling centers in Iran, people looked for nicotine replacement therapies. Nass has nicotine and can alleviate the symptoms of smoking cessation; hence people started using nass instead of smoking cigarettes.

The use of smokeless tobacco to quit smoking has also been reported in other places. In Sweden, a quarter of men who decided to quit smoking used Snus^14,15^. Furthermore, using Snus has been reported to reduce cigarette smoking among Swedish men and women from 1970 to 2000^16^. Norberg et al.^17^ reported that between 1990 and 2007, the prevalence of smoking among men and women significantly decreased.

According to the participants of the current study, drug addiction also contributes to nass consumption. Drug users believed that using nass after taking a drug doubled or even tripled the effect of the drug. They also believed that using nass prolongs the effect of the drug. Also, since drugs dry the user’s mouth, nass consumed after drug use does not melt quickly and stays in the mouth for much longer.

Certain occupations, such as fishing, driving and military service, were referred to as predisposing factors of nass consumption by some participants. The majority of people in the cities and villages by the Caspian Sea, such as Turkmen and Ghomishan, are fishermen and they often fish illegally, i.e. without having a license. Cigarette lights can be seen from far away and give away the location of the fishermen. Therefore, those who smoke cigarettes while fishing illegally are always in danger of being spotted and caught by the Coast Guards. To avoid this, these fishermen use nass instead of smoking cigarettes. Moreover, soldiers usually served their mandatory military service at places far away from their home. Mandatory military service is known to be a very difficult time for soldiers. Many of these soldiers started nass consumption to cope with the stress, calm their nerves, avoid cold weather, and simply stay awake while being on guard at night shifts. Unfortunately, by the time these soldiers completed their military service and returned home, nass consumption had become a well-stablished habit.

A small group of participants claimed that truck drivers have encouraged them to consume nass. When driving a truck to harvest crops, such as barley and wheat during the warm months of June, July and August, people preferred to use nass instead of cigarettes to prevent the potential danger of fire caused by careless use of cigarettes or improperly discarded cigarette butts.

The results of this study showed that people’s beliefs about nass, particularly those related to its health benefits, were also among the main factors contributing to nass consumption. In some areas, especially the rural areas where there were fewer medical facilities, the elderly recommended use of nass for pain relief, e.g. to alleviate toothache. Furthermore, some people, especially those with heavy and strenuous manual jobs, believed that nass consumption could increase their physical strength. Therefore, they started using nass to boost their physical strength. They also believed that nass consumption would save them time at their workplace since, unlike cigarette smoking, they did not have to stop working to use nass. In Myanmar, tobacco-free betel was originally used to cover up bad breath and also help digestion; later on tobacco was added to betel^10^.

### Limitations

This was the first qualitative study that explored the factors contributing to nass consumption among people in Turkmen-Sahra. In this study, individual interviews and focus group discussions were conducted in natural environments and social contexts. The participants were selected from 4 cities with higher prevalence of nass consumption. They were from different age groups and had different occupations, allowing the researchers to achieve a deeper understanding of the factors that encouraged use of nass. However, the study had a number of limitations. Although nass consumers are generally males, lack of female participants was one of the limitations of this study. Furthermore, future studies should consider recruiting participants from towns such as Maraveh-tapeh and Kalaleh where the prevalence of nass consumption is lower and also from Turkmen living in the province of North Khorasan.

### Implications for policies and research

Findings of the present study are useful for health policy makers in planning and implementing intervention strategies to prevent nass consumption. Nass consumption prevention strategies in Turkmen society should focus on teenagers, a population at greater risk. Additionally, the results support the fact that easy access to nass is one of the main factors contributing to its consumption and hence should be given serious consideration when planning preventive strategies. Moreover, strict rules and policies should be created and implemented to regulate and control nass production and its distribution and sale in the society. Establishing ‘stop smoking’ clinics should also be considered to help those who want to quit smoking and prevent them from switching to nass.

## CONCLUSIONS

The results of the present study showed the following as the main reasons for nass consumption among Turkmen: 1) cultural, social, and environmental facilitators, 2) nass was considered as an alternative to cigarette, 3) nass was believed to intensify the effects of opium and other drugs, 4) specific occupations and circumstances, and 5) beliefs related to nass. These findings could be valuable for health policy makers in supporting the prevention of nass consumption based on the behavioral triangle of enabling, strengthening and facilitating factors presented by Green and Kruter^18^. Health policy makers henceforth should plan and implement preventive strategies for nass consumption.

## References

[1] Critchley JA, Unal B (2003). Health effects associated with smokeless tobacco: a systematic review. Thorax.

[2] Bahreinifar S, Sheon NM, Ling PM (2013). Is snus the same as dip? Smokers’ perceptions of new smokeless tobacco advertising. Tobacco Control.

[3] Maki J (2015). The incentives created by a harm reduction approach to smoking cessation: Snus and smoking in Sweden and Finland. International Journal of Drug Policy.

[4] Henley SJ, Thun MJ, Connell C, Calle EE (2005). Two large prospective studies of mortality among men who use snuff or chewing tobacco (United States). Cancer Causes and Control.

[5] Roland M, Asma S, Backinger C (2002). Smokeless tobacco fact sheets.

[6] Statistical center of Iran General Population and Housing Census Iran 2016.

[7] Islami F, Pourshams A, Vedanthan R (2012). Smoking water-pipe, chewing nass and prevalence of heart disease: a cross-sectional analysis of baseline data from the Golestan Cohort Study, Iran. Heart.

[8] Woods P (2006). Successful writing for qualitative researchers.

[9] Frey JH, Fontana A (1991). The group interview in social research. The Social Science Journal.

[10] Sein T, Swe T, Toe MM, Zaw KK, Sein TO (2014). Challenges of smokeless tobacco use in Myanmar. Indian Journal of Cancer.

[11] Akl EA, Jawad M, Lam WY, Co CN, Obeid R, Irani J (2013). Motives, beliefs and attitudes towards waterpipe tobacco smoking: a systematic review. Harm Reduction Journal.

[12] Afifi R, Khalil J, Fouad F (2013). Social norms and attitudes linked to waterpipe use in the Eastern Mediterranean Region. Social Science & Medicine.

[13] Harakeh Z, Vollebergh WA (2012). The impact of active and passive peer influence on young adult smoking: An experimental study. Drug and Alcohol Dependence.

[14] Ramström L, Foulds J (2006). Role of snus in initiation and cessation of tobacco smoking in Sweden. Tobacco Control.

[15] Gilljam H, Galanti MR (2003). Role of snus (oral moist snuff) in smoking cessation and smoking reduction in Sweden. Addiction.

[16] Foulds J, Ramstrom L, Burke M, Fagerström K (2003). Effect of smokeless tobacco (snus) on smoking and public health in Sweden. Tobacco Control.

[17] Norberg M, Lundqvist G, Nilsson M, Gilljam H, Weinehall L (2011). Changing patterns of tobacco use in a middle-aged population - the role of snus, gender, age, and education. Global Health Action.

[18] Green L, Kreuter M (1999). The precede-proceed model. Health promotion planning: an educational approach.

